# Correction: Glucocorticoid receptor-mediated amygdalar metaplasticity underlies adaptive modulation of fear memory by stress

**DOI:** 10.7554/eLife.103529

**Published:** 2024-09-26

**Authors:** Ran Inoue, Kareem Abdou, Ayumi Hayashi-Tanaka, Shin-ichi Muramatsu, Kaori Mino, Kaoru Inokuchi, Hisashi Mori

**Keywords:** Mouse

Inoue R, Abdou K, Hayashi-Tanaka A, Muramatsu SI, Mino K, Inokuchi K, Mori H. 2018. Glucocorticoid receptor-mediated amygdalar metaplasticity underlies adaptive modulation of fear memory by stress. *eLife* **7**:e34135. doi: 10.7554/eLife.34135.

Published 26 June 2018

A reader brought to our attention the duplication of a trace that appears in our eLife paper and also in an earlier 2018 Science Report article (Abdou et al., 2018, https://doi.org/10.1126/science.aat3810). We carefully checked the published eLife article and found two mistakenly used figures, Figure 6F and 6G, which represent the LTP and LTD traces for Floxed GR mice, respectively.

The recordings from the Master 8-pClamp system for both papers (eLife 2018 and Science 2018) are stored in separate folders, ensuring that there is no mixing of data. The only shared element between the two publications is the Illustrator (.ai) file used to generate the high-resolution figures. Typically, a template file is reused after deleting its previous content to serve as a guide for the ideal size of each panel. We believe that during the process of replacing the traces for the Science paper with those for the eLife paper, we inadvertently forgot to replace two of the traces for the Floxed GR mice.

We are therefore correcting Figure 6F and 6G with the raw data representative traces for Floxed GR mice. These corrections do not affect the data in Figure 6 nor impact the relevant results and conclusions.

The corrected Figure 6 is shown here:

**Figure 1:**
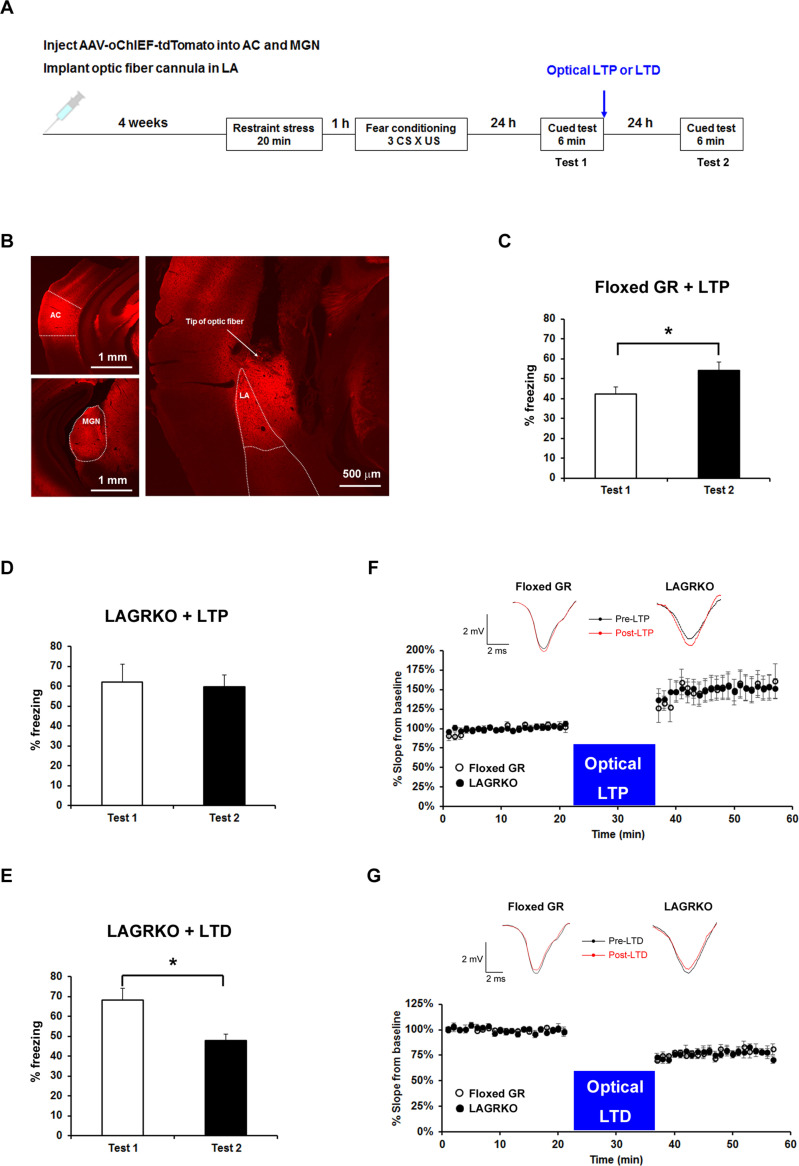


The originally published Figure 6 is shown for reference:

**Figure 2:**
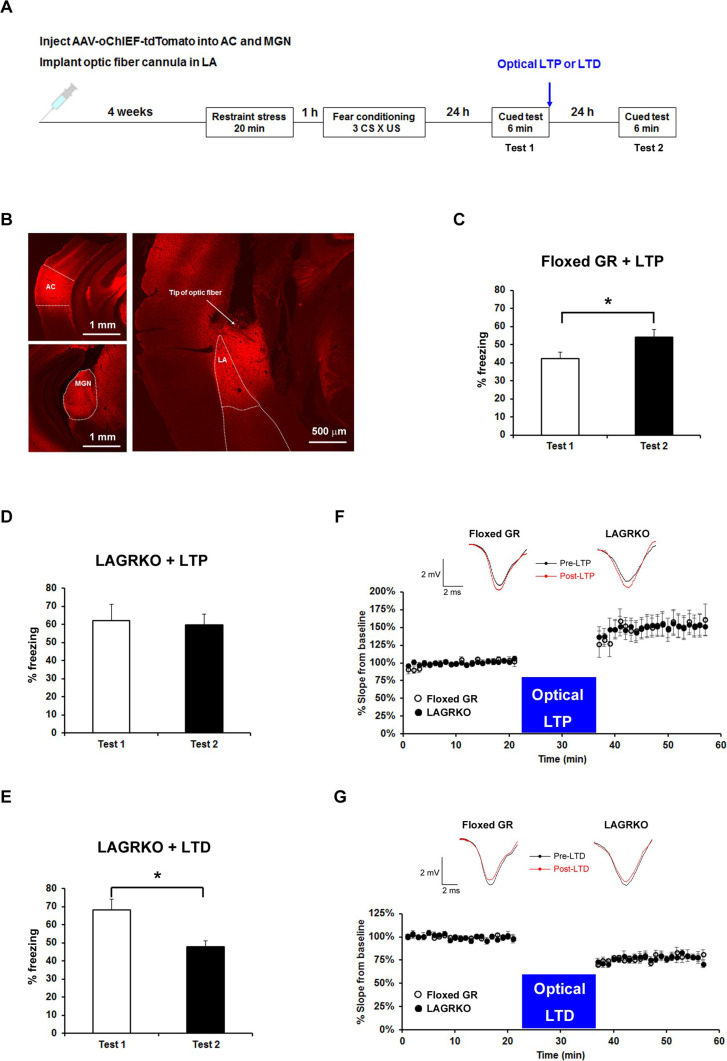


The article has been corrected accordingly.

